# Determination of basal ileal endogenous losses and standardized ileal digestibility of amino acids in barley fed to growing pigs

**DOI:** 10.1186/s40104-016-0115-7

**Published:** 2016-09-26

**Authors:** Hanna Katharina Spindler, Rainer Mosenthin, Pia Rosenfelder, Henry Jørgensen, Knud Erik Bach Knudsen, Meike Eklund

**Affiliations:** 1 University of Hohenheim, Institute of Animal Science, Emil-Wolff-Strasse 10, 70599 Stuttgart, Germany; 2 Aarhus University, Department of Animal Science, Research Center Foulum, 8830 Tjele, Denmark

**Keywords:** Amino acid, Barley, Basal ileal endogenous loss, Growing pigs, Regression analysis, Standardized ileal digestibility

## Abstract

**Background:**

Basal ileal endogenous amino acid (AA) losses (IAA_end_) and standardized ileal digestibility (SID) values of cereal grains, such as barley, are apparently underestimated when determined according to the nitrogen (N)-free method. Regression analysis between the dietary apparent ileal digestible content (cAID) and total crude protein (CP) and AA can be considered as alternative approach to obtain more accurate values for IAA_end_ and SID of AA in cereal grains.

**Methods:**

Eight hulled barley genotypes were used, with barley being the only source of CP and AA in the assay diets. The diets contained 95 % as-fed of these eight barley genotypes each, ranging in CP content between 109.1 and 123.8 g/kg dry matter (DM). Nine ileally T-cannulated barrows, average body weight (BW) 30 ± 2 kg, were allotted to a row-column design comprising eight periods with 6 d each and nine pigs. On d 5 and the night of d 6 of every period, ileal digesta were collected for a total of 12 h. The IAAend and the SID were determined by linear regression analysis between cAID and total dietary CP and AA.

**Results:**

There exist linear relationships between cAID and total CP and AA (*P* < 0.001). The IAAend of CP, Lys, Met, Thr and Trp amounted to 35.34, 1.08, 0.25, 1.02 and 0.38 g/kg DM intake (DMI), respectively, which are greater compared to average IAAend determined previously under N-free feeding conditions. The SID of CP, Lys, Met, Thr and Trp was 90, 79, 85, 79 and 86 %, respectively, and was greater when compared to tabulated values. Moreover, these SID values were greater than those reported in literature, based on correction of apparent ileal digestibility (AID) of CP and AA for their IAA_end_ values. Summarized, the results of the present regression analysis indicate greater IAA_end_ in barley-based diets compared to those obtained by N-free feeding.

**Conclusions:**

For low-protein feed ingredients like barley the regression method may be preferred over correction of AID values for their IAA_end_ determined under N-free feeding conditions, as intercepts and slopes of the linear regression equations between cAID and total dietary CP and AA provide direct estimates of IAA_end_ and SID of CP and AA in the presence of the assay feed ingredient.

## Background

About 308 million tons of cereals are annually harvested in the European Union [1]. Despite their low crude protein (CP) content, they can supply more than half of the feed protein in pig diets due to their high dietary inclusion level [2]. Among different genotypes of the same cereal exists considerable variation both in contents and in standardized ileal digestibility (SID) of CP and amino acids (AA). For example, large standard deviations for SID of CP and Lys of 13.0 and 10.6 %, respectively, have been reported for barley [3]. Besides chemical composition, especially non-starch-polysaccharides (NSP), which may affect SID of CP and AA in cereals [4, 5], differences in basal ileal endogenous loss of CP and AA (IAA_end_) between different studies have been identified for being one of the major factors responsible for the observed variation in SID of CP and AA in cereals including barley [3].

The use of N-free diets has been suggested as a routine procedure to generate SID values in feed ingredients [6]. With this method, Spindler et al. [7] determined relatively low SID of AA in barley amounting only to 74 % for CP, and 67, 79, 73 and 75 % for Lys, Met, Thr and Trp, respectively, compared to current nutrient tables. Nitrogen-free feeding, however, has in some cases been criticized to be not physiological, thereby resulting in smaller estimates of IAA_end_ [8] when compared with the regression analysis method for determination of IAA_end_ in low-protein assay diets e.g. based on rye [9], triticale [10] or wheat [11]. Moreover, at low dietary CP and AA levels, such as in diets containing cereal grains as sole dietary CP source, correction of AID for IAA_end_ may result in quite variable SID of CP and AA [12, 13] due to a large and varying proportion of IAA_end_ to total ileal CP and AA recovery. Regression analysis between the apparent ileal digestible and total dietary CP and AA content has been proposed to provide direct estimates of IAA_end_ and SID of AA in the presence of graded dietary CP and AA levels from the assay feed ingredient [3, 12].

The regression method used in the present study allows for estimating IAA_end_ in pigs in the presence of eight barley genotypes different in CP, by extrapolating the dietary CP and AA intakes to zero intake, and at the same time, estimates of SID of AA in barley can be derived from the slope of the regression analysis.

## Methods

### Animal, diets and experimental design

Nine crossbred barrows (Duroc × Landrace × Yorkshire), initial BW of 30 ± 2 kg, were housed individually in smooth sided pens with a concrete floor (area of 2.8 m^2^), and floor heating. The room was temperature-controlled (22 ± 2 °C). Water was available at all times. After 2 wk of adaption, the pigs were surgically fitted with a simple T-cannula at the distal ileum. The pigs were allowed six to seven d of recuperation. Body weight of the pigs at the end of the experiment was 64 ± 4 kg. The animals were allotted to an 8 × 9 row-column design with eight periods of 6 d each and nine pigs.

Eight hulled winter barley genotypes, including Campanile (**B1**; LIMAGRAIN GmbH, Edemissen, Germany), Yool (**B2**; Syngenta Seeds GmbH, Bad Salzuflen, Germany), Lomerit (**B3**; KWS LOCHOW GmbH, Bergen, Germany), Travira (**B4**; Ackermann Saatzucht GmbH & Co. KG, Irlbach, Germany), Anisette (**B5**; NORDSAAT Saatzucht-GmbH, Langenstein, Germany), Fridericus (**B6**; KWS LOCHOW GmbH, Bergen, Germany), Canberra (**B7**; LIMAGRAIN GmbH, Edemissen, Germany), and Metaxa (**B8**; Ackermann Saatzucht GmbH & Co. KG, Irlbach, Germany) were used. The eight assay diets contained one of the eight barley genotypes each, thus barley was the only source of dietary CP and AA (Table 1). All diets had the same inclusion level of barley (951.3 g/kg as-fed). The barley batches were ground using a hammer mill (SKIOLD A/S, Sæby, Denmark) to pass a 2-mm sieve. A commercial mineral and vitamin premix, monocalcium phosphate, sodium chloride, and calcium carbonate were added to the assay diets to meet the NRC [14] nutrient requirements for pigs above 20 kg. All assay diets were supplemented with plant oil at a level of 20 g/kg (as-fed) to reduce the dustiness of the diets, and 0.2 g/kg (as-fed) of dl-*α*-tocopherol acetate was added as antioxidant. Titanium dioxide (7 g/kg) was used as an indigestible marker. At the beginning of every period, the pigs where weighed and fed at a daily level of 40 g/kg (as-fed) of average BW of all animals, corresponding to approximately 3 times their daily energy requirement for maintenance (i.e. 106 kcal of ME (as-fed)/kg of BW^0.75^; [14]).

**Table 1:** Ingredient composition of the assay diets (g/kg, as-fed)

Item	Assay diets
Barley^a^	951.3
Oil^b^	20.0
Vitamins and mineral mix^c^	16.2
Monocalcium phosphate	3.5
Calcium carbonate	1.8
Vitamin E^d^	0.2
Titanium dioxide	7.0

^a^ *n* = eight genotypes of barley each

^b^Blend of rapeseed (75 %) and soybean oil (25 %)

^c^Deutsche Vilomix Tierernährung GmbH, Neuenkirchen-Vörden, Germany. Supplied per kg of diet: Ca, 4.0 g; P, 0.8 g; Na, 0.9 g; Mg, 162 mg; Fe, 65 mg (FeSO_4_ · H_2_O); Cu, 8 mg (CuSO_4_ · 5H_2_O); Mn, 43 mg (MnO); Zn, 54 mg (ZnO); J, 1.1 mg (Ca(IO_3_)_2_); Se, 0.2 mg (Na_2_SeO_3_); Co, 0.1 mg (2CoCO_3_ · 3Co(OH)_2_ · H_2_O); Vitamin A, 6480 IU; Vitamin D_3_, 972 IU; Vitamin E, 41 mg; Vitamin B_1_, 1 mg; Vitamin B_2_, 3 mg; Vitamin B_6_, 2 mg; Vitamin B_12_, 16 μg; Vitamin K_3_, 2 mg; Niacin, 10 mg; Ca-pantothenat, 6 mg; Folic acid, 0.4 mg; Choline chloride, 122 mg

^d^Euro OTC PHARMA GmbH, Bönen, Germany

### Experimental feeding and sample collection

Feed was divided into two equal meals, and fed to pigs at 0800 and 2000 h. The initial 4 d of every period were considered as an adaption period to the assay diets. During the initial 3.5 d of every period, a crystalline AA mixture (l-Gly, 775.1 g/kg as-fed; l-Lys-HCl, 106.4 g/kg as-fed; dl-Met, 15.9 g/kg as-fed; l-Thr, 29.1 g/kg as-fed; l-Trp, 5.1 g/kg as-fed; l-Ile, 18.2 g/kg as-fed; l-Leu, 25.7 g/kg as-fed; l-Val, 16.0 g/kg as-fed; l-His, 6.5 g/kg as-fed; l-Phe, 2.0 g/kg as-fed; the total amount of the AA mixture was adjusted to the daily feed allowance, and amounted to 50, 54, 59, 64, 69, 75, 81 and 87 g/feeding in periods one to eight, respectively) was added on top of the assay diets to attenuate the effect of feeding low-protein diets [15]. At the evening of d 4, 2 g/kg of chromic oxide was added to the assay diets to visually differentiate digesta originating from the assay diets without synthetic AA by their green color. Digesta was collected for a total of 12 h on d 5 of period one, three, five, and seven from 1000 to 1200, 1400 to 1600, and 1800 to 2000 h, and on d 6 of period one, three, five, and seven from 2000 to 2200, 2400 to 0200, and 0400 to 0600 h. Additionally, digesta collection was conducted on d 5 of period two, four, six, and eight from 0800 to 1000, 1200 to 1400, and 1600 to 1800 h, and on d 6 of period two, four, six, and eight from 2200 to 2400, 0200 to 0400, and 0600 to 0800 h. Ileal digesta were collected in plastic bags. Bags were removed at least every 30 min, and 4 mL of 2.5 mol/L formic acid were added to every bag to minimize further bacterial fermentation. Digesta samples were pooled within pig and period and immediately frozen at −20 °C.

### Chemical analyses

Digesta samples were freeze-dried, ground to 0.5 mm, and homogenized before analyses. All chemical analyses were conducted as described by Rosenfelder et al. [16]. Methods of VDLUFA [17] were used to determine contents of dry matter (DM) in barley and in ileal digesta samples (method 3.1). Accordingly, ash content in barley was measured based on method 8.1, CP in barley with method 4.1.1, neutral detergent fiber (NDF) was assayed with heat-stable amylase exclusive of residual ash (aNDFom; method 6.5.1), and acid detergent fiber (ADF), also expressed exclusive of residual ash, (ADFom) according to method 6.5.2. Acid detergent lignin (ADL) was determined by solubilization of cellulose with sulphuric acid (ADL (sa); method 6.5.3), and starch according to the polarimetric method (7.2.1).

Non-starch polysaccharides, and Klason lignin in barley were determined by enzymatic-colorimetric and enzymatic-chemical-gravimetric methods as described by Bach Knudsen [18]. Klason lignin was determined by a two-stage sulfuric acid hydrolysis, and is supposed to be more accurate in estimating plant cell-wall lignin content than ADL [19–21]. Content of total and soluble arabinoxylan was determined as the sum of arabinose and xylose residues in total and soluble NSP. Total and insoluble *β*-glucans were analyzed by the enzymatic-colorimetric method of McCleary and Glennie-Holmes [22]. Soluble *β*-glucan was calculated as difference between total *β*-glucan and insoluble *β*-glucan after extraction of the soluble *β*-glucan in water [23].

Gross energy (GE) content in the eight barley genotypes was measured using a bomb calorimeter (IKA calorimeter, C200, IKA®-Werke GmbH & Co. KG, Staufen, Germany). Nitrogen (N) contents in the eight assay diets, and digesta samples were analyzed using a gas combustion method according to the official method 990.03 of the AOAC [24]. Crude protein content was calculated by multiplying the content of N with 6.25. Ethlyenediaminetetraacetic acid was used as a reference standard before and after all N analyses. Amino acid contents in the barley genotypes, assay diets and digesta samples were determined by using ion-exchange chromatography with postcolumn derivatization with ninhydrin. The AA were oxidized with performic acid which was neutralized by sodium metabisulfite [25, 26]. They were hydrolyzed by means of 6 mol/L HCl for 24 h at 110 °C. Thereafter, they were quantified with the internal standard method (norleucine) by measuring the absorption of reaction products with ninhydrin at 570 nm. Tryptophan was determined by HPLC with fluorescence detection (extinction 280 nm, emission 356 nm) after alkaline hydrolysis with barium hydroxide octahydrate for 20 h at 110 °C [27]. Concentrations of titanium dioxide in the assay diets and the ileal digesta samples were determined according to a method outlined by Brandt and Allam [28].

### Calculations

The apparent ileal digestible content (cAID) of CP and AA in the assay diets were calculated according to the equation:

1 $$ \mathrm{cAID}=\mathrm{A}{\mathrm{A}}_{\mathrm{diet}}-\left[\left(\mathrm{T}{\mathrm{i}}_{\mathrm{diet}}\times \mathrm{A}{\mathrm{A}}_{\mathrm{digesta}}\right)/\mathrm{T}{\mathrm{i}}_{\mathrm{digesta}}\right] $$

where cAID = apparent ileal digestible content of CP or AA in the assay diet (g/kg DMI); AA_diet_ = CP or AA content in the assay diet (g/kg DMI); Ti_diet_ = marker content in the assay diet (g/kg DM); AA_digesta_ = CP or AA content in ileal digesta (g/kg DMI), and Ti_digesta_ = marker content in ileal digesta (g/kg DM).

For determination of IAA_end_ and SID of CP and AA, a simple linear regression model was used. The relationship between SID and apparent ileal digestibility (AID) of CP and AA in the assay diet can either be expressed by Eq. (2) or, after further mathematical derivation, by Eq. (3):

2 $$ \mathrm{S}\mathrm{I}\mathrm{D}=\left[\left(\mathrm{cAID}/\mathrm{A}{\mathrm{A}}_{\mathrm{diet}}\right)+\left(\mathrm{I}\mathrm{A}{\mathrm{A}}_{\mathrm{end}}/\mathrm{A}{\mathrm{A}}_{\mathrm{diet}}\right)\right] $$

3 $$ \mathrm{cAID}=-\mathrm{I}\mathrm{A}{\mathrm{A}}_{\mathrm{end}}+\left[\left(\mathrm{S}\mathrm{I}\mathrm{D}/100\right)\times \mathrm{A}{\mathrm{A}}_{\mathrm{diet}}\right] $$

where SID = standardized ileal digestibility of CP or AA in the assay diet and barley; cAID = cAID of CP or AA in assay diet, determined using Eq. 1; AA_diet_ = total content of CP or AA in the assay diet (g/kg DMI); IAA_end_ = basal ileal endogenous CP and AA losses (g/kg DMI). Contents of apparent ileal digestible CP and AA are dependent, and AA_diet_ are independent variables [12]. The regression coefficients IAA_end_ and SID of CP and AA are estimated by fitting the linear regression model. If relationships between cAID and total content of CP and AA in the assay diets are linear, dietary CP and AA intakes can be extrapolated to zero intake for determination of IAA_end_. Furthermore, SID in the assay diets and barley is represented by the slope of the linear regression model [3, 13].

### Statistical analysis

Linear regression analysis between cAID of CP and AA and the dietary CP and AA content was used to test homogeneity of variances, normal distribution of the data and to detect outliners by analysis of the studentized residuals using the UNIVARIATE procedure of SAS [29]. The cAID of CP and AA of every assay diet were analyzed using the MIXED procedure of SAS. The results on cAID of CP and AA were reported as least squares means. Linear and quadratic regression analyses between dietary apparent ileal digestible and total CP and AA contents were conducted, and the best structure according to the Akaike Information Criterion was selected. Thereafter, linear regression analyses between cAID and total CP and AA in the diets were performed, to determine IAA_end_ and SID of CP and AA in barley batches. All models included the fixed effect of dietary CP and (or) AA level and animal, whereas period and period × pig were considered random effects. Linear regression analyses were performed on effects of chemical composition on SID of CP and AA. For all Wald-type F-tests the significance level was set at α = 0.05.

## Results

### General observations

All pigs remained healthy and readily consumed their daily feed allowances. Occasional feed refusals were recorded. A total of four outliers were detected across all eight dietary treatments, and these observations were therefore removed from the data set before further statistical analyses. Finally, a total of 68 observations were included in the model for estimation of IAA_end_ and determination of SID of CP and AA in barley genotypes.

### Chemical composition of the barley genotypes and contents of apparent ileal digestible CP and AA in the barley-based assay diets

The chemical composition of the eight barley genotypes is shown in Table 2. Their contents of CP, Lys, Met, Thr, and Trp ranged from 112.6 to 129.2, 4.0 to 4.5, 1.8 to 2.1, 3.9 to 4.3, and 1.4 to 1.6 g/kg DM, respectively, whereas contents of starch, aNDFom, ADFom, ADL (sa), and NSP varied from 533.0 to 564.0, 180.2 to 209.1, 44.5 to 63.4, < 5.0 to 8.9, and 168.0 to 184.1 g/kg DM, respectively. All diets had the same inclusion level of barley, and due to the different CP contents in the eight barley genotypes, CP contents increased from 109.1 to 123.8 g/kg DM from assay diets B1 to B8 (Table 3). Contents of apparent ileal digestible AA (g/kg of DMI) in the assay diets are presented in Table 4. In response to the increase in dietary CP level from 109 to 124 g/kg DM, the apparent ileal digestible CP and AA contents linearly increased (*P* < 0.01, except for Gly (*P* < 0.05)). For some AA, there were also quadratic increases, however, the linear regression model fitted in all cases better than the quadratic one, which was reflected in smaller Akaike Information Criterion values in the linear compared to the quadratic model (data not shown).

**Table 2:** Chemical composition of the eight barley genotypes (g/kg dry matter)

Item	B1	B2	B3	B4	B5	B6	B7	B8
CP	118.9	112.6	117.3	116.6	127.4	129.2	125.9	128.4
Indispensable AA								
Arg	6.0	5.7	5.8	6.1	6.2	6.5	6.5	6.3
His	2.6	2.5	2.6	2.6	2.6	2.9	2.6	2.6
Ile	4.3	3.9	4.2	3.8	4.5	4.4	4.2	4.2
Leu	8.1	7.7	7.7	8.0	8.7	8.9	8.9	8.9
Lys	4.1	4.0	4.0	4.5	4.1	4.4	4.5	4.3
Met	1.9	1.8	1.9	1.8	2.1	2.1	2.1	2.1
Phe	5.7	5.7	5.7	5.6	6.8	6.5	6.4	6.9
Thr	3.9	3.9	3.9	4.1	4.2	4.3	4.2	4.2
Trp	1.4	1.5	1.4	1.5	1.6	1.6	1.6	1.6
Val	5.9	5.5	5.9	5.7	6.2	6.3	6.1	5.9
Dispensable AA								
Ala	4.7	4.4	4.7	4.9	4.8	5.0	4.9	4.9
Asp	7.1	6.7	6.7	7.2	7.1	7.5	7.4	7.2
Cys	2.4	2.5	2.5	2.7	2.6	2.7	3.0	2.9
Glu	28.6	26.3	28.4	26.1	31.9	31.8	29.6	30.9
Gly	4.6	4.8	4.7	5.1	4.8	5.5	5.1	5.0
Pro	12.8	11.7	12.5	11.5	14.6	14.5	13.4	14.4
Ser	5.1	4.9	5.0	5.1	5.5	5.5	5.5	5.4
Dry matter	879.0	878.0	880.0	877.0	879.0	881.0	882.0	882.0
Gross energy MJ/kg DM	18.7	18.7	18.7	18.7	18.8	19.1	17.7	18.8
Starch	560.0	564.0	545.0	548.0	541.0	533.0	546.0	550.0
*β*-glucan	39.5	50.3	43.9	45.7	53.3	43.3	49.9	48.2
Soluble *β*-glucan	20.6	27.0	20.3	24.8	25.8	24.4	26.6	23.5
Arabinoxylan	81.1	74.4	75.5	75.7	70.5	76.6	86.4	78.8
Soluble arabinoxylan	7.2	4.0	6.5	10.4	7.4	7.1	20.1	14.7
Total NSP	171.1	168.0	176.7	167.0	168.3	168.4	184.1	172.7
Soluble NSP	40.3	46.1	50.5	49.9	55.4	37.6	66.1	58.6
Klason lignin	26.9	20.1	20.7	15.2	24.5	27.4	22.9	18.9
aNDFom	180.2	207.7	209.1	205.3	191.1	194.1	181.4	182.5
ADFom	52.6	51.3	56.3	50.5	44.5	63.4	52.9	46.2
ADL (sa)	8.9	8.7	<0.5	8.0	4.8	7.9	8.5	6.6

*CP* crude protein, *AA* amino acids, *NSP* non-starch polysaccharides, *aNDF* neutral detergent fiber with heat stable amylase and expressed exclusive residual ash, *ADFom* acid detergent fiber expressed exclusive of residual ash, *ADL (sa)* acid detergent lignin determined by solubilization of cellulose with sulphuric acid

**Table 3:** Contents of crude protein (CP) and amino acids (AA) in the barley-based assay diets (g/kg dry matter)

Item	B1	B2	B3	B4	B5	B6	B7	B8
CP	109.1	109.6	109.6	114.2	117.5	120.4	122.1	123.8
Indispensable AA								
Arg	5.5	5.9	5.1	5.6	6.0	5.9	5.9	6.1
His	2.4	2.5	2.2	2.4	2.4	2.6	2.6	2.5
Ile	3.5	3.7	3.7	4.0	3.9	3.8	4.4	4.0
Leu	7.4	7.8	6.9	7.6	8.2	8.1	8.4	8.6
Lys	4.0	4.1	3.5	3.8	4.1	4.1	4.0	4.2
Met	1.7	1.8	1.7	1.8	1.8	1.8	2.0	1.9
Phe	5.1	5.6	4.9	5.3	5.9	6.2	6.2	6.6
Thr	3.7	3.9	3.6	3.6	3.9	4.1	4.0	4.1
Trp	1.5	1.5	1.4	1.4	1.6	1.6	1.6	1.6
Val	5.3	5.4	5.0	5.4	5.4	5.5	6.1	5.7
Dispensable AA								
Ala	4.4	4.6	4.1	4.4	4.5	4.6	4.6	4.7
Asp	6.5	6.9	5.9	6.5	6.9	6.9	6.8	6.9
Cys	2.5	2.5	2.2	2.3	2.7	2.6	2.6	2.8
Glu	24.1	26.1	26.0	27.1	26.7	28.4	30.1	29.5
Gly	4.6	4.8	4.1	4.3	4.6	5.0	4.5	4.7
Pro	10.9	11.7	11.5	12.0	12.5	13.2	13.9	14.1
Ser	4.6	4.8	4.6	4.9	5.0	5.1	5.1	5.2

**Table 4:** Contents of apparent ileal digestible crude protein (CP) and amino acids (AA; LSMeans ± SEM, g/kg dry matter intake) in barley-based assay diets fed to growing pigs

Item	B1	B2	B3	B4	B5	B6	B7	B8	*P*-value*
n^a^	9	9	9	7	8	9	8	9	
CP	65.2 ± 1.18	72.9 ± 1.17	69.1 ± 1.17	62.4 ± 1.18	74.8 ± 1.18	73.9 ± 1.18	80.0 ± 1.17	76.6 ± 1.09	<0.001
Indispensable AA									
Arg	4.2 ± 0.05	4.1 ± 0.05	3.7 ± 0.05	3.9 ± 0.05	4.2 ± 0.05	4.5 ± 0.05	4.6 ± 0.05	4.2 ± 0.04	<0.001
His	1.2 ± 0.02	1.7 ± 0.02	1.6 ± 0.02	1.6 ± 0.02	1.8 ± 0.02	1.7 ± 0.02	1.8 ± 0.02	1.8 ± 0.02	<0.001
Ile	2.4 ± 0.03	2.7 ± 0.03	2.5 ± 0.03	2.1 ± 0.03	3.0 ± 0.03	2.6 ± 0.03	2.6 ± 0.03	2.5 ± 0.03	<0.001
Leu	5.4 ± 0.06	5.4 ± 0.06	4.9 ± 0.06	5.0 ± 0.06	5.9 ± 0.06	5.9 ± 0.06	6.2 ± 0.06	5.8 ± 0.06	<0.001
Lys	2.2 ± 0.05	2.1 ± 0.05	1.8 ± 0.05	2.0 ± 0.05	2.1 ± 0.05	2.3 ± 0.05	2.3 ± 0.05	2.2 ± 0.05	<0.001
Met	1.3 ± 0.01	1.3 ± 0.01	1.2 ± 0.01	1.1 ± 0.01	1.5 ± 0.01	1.3 ± 0.01	1.4 ± 0.01	1.3 ± 0.01	<0.001
Phe	3.9 ± 0.05	3.7 ± 0.05	3.4 ± 0.05	3.3 ± 0.05	4.4 ± 0.05	4.2 ± 0.05	4.8 ± 0.05	4.5 ± 0.05	<0.001
Thr	2.2 ± 0.05	1.9 ± 0.05	2.0 ± 0.05	1.9 ± 0.05	2.1 ± 0.05	2.1 ± 0.05	2.3 ± 0.05	2.3 ± 0.05	<0.001
Trp	0.8 ± 0.02	0.8 ± 0.02	0.7 ± 0.02	0.8 ± 0.02	0.9 ± 0.02	0.9 ± 0.02	0.9 ± 0.02	0.9 ± 0.01	<0.001
Val	3.5 ± 0.05	3.7 ± 0.05	3.4 ± 0.05	3.4 ± 0.05	4.2 ± 0.05	3.7 ± 0.05	3.8 ± 0.05	3.7 ± 0.04	<0.001
Dispensable AA									
Ala	2.5 ± 0.06	2.4 ± 0.06	2.2 ± 0.06	2.2 ± 0.06	2.4 ± 0.06	2.5 ± 0.06	2.7 ± 0.06	2.5 ± 0.06	<0.001
Asp	3.9 ± 0.08	3.7 ± 0.08	3.1 ± 0.08	3.4 ± 0.08	3.6 ± 0.08	4.0 ± 0.08	3.9 ± 0.08	3.8 ± 0.08	<0.001
Cys	1.7 ± 0.02	1.6 ± 0.02	1.6 ± 0.02	1.7 ± 0.02	1.8 ± 0.02	2.0 ± 0.02	2.1 ± 0.02	1.9 ± 0.02	<0.001
Glu	20.6 ± 0.15	22.4 ± 0.14	21.6 ± 0.14	18.7 ± 0.15	25.1 ± 0.15	21.6 ± 0.15	24.1 ± 0.14	23.4 ± 0.13	<0.001
Gly	2.2 ± 0.10	1.8 ± 0.10	1.7 ± 0.10	2.0 ± 0.10	1.8 ± 0.10	2.2 ± 0.10	2.3 ± 0.10	2.5 ± 0.09	0.025
Pro	8.1 ± 0.22	8.8 ± 0.22	8.5 ± 0.22	7.4 ± 0.22	10.2 ± 0.22	9.1 ± 0.22	10.8 ± 0.22	9.8 ± 0.20	<0.001
Ser	3.1 ± 0.06	3.2 ± 0.05	3.0 ± 0.05	2.8 ± 0.05	3.3 ± 0.05	3.3 ± 0.05	3.5 ± 0.05	3.3 ± 0.05	<0.001

*P-value for linear effect

^a^ *n* = number of pigs per treatment

### Basal ileal endogenous losses and standardized ileal digestibility of CP and AA in the barley-based assay diets

Due to significant linear relationships between contents of apparent ileal digestible and total CP and AA in the assay diets (*P* < 0.001), both IAA_end_, and SID of CP and AA in the barley genotypes can be derived from the results of the regression analysis. Moreover, intercepts and slopes of the linear regression equations provide direct estimates of IAA_end_ and SID of CP and AA (Table 5). All estimated IAA_end_ were significantly different from zero (*P* < 0.05). Basal ileal endogenous loss of CP and AA, obtained by extrapolating dietary CP and AA levels to zero intake, was 35.34, 1.08, 0.25, 1.02, and 0.38 g/kg DMI for CP, Lys, Met, Thr, and Trp respectively. Overall, IAA_end_ ranged from 0.25 g/kg DMI (Met) to 1.29 g/kg DMI (Leu) for indispensable and from 0.43 g/kg DMI (Cys) to 3.44 g/kg DMI (Glu) for dispensable AA. The slopes of all linear regression equations which represent SID values for CP and AA, were significant (*P* < 0.001) and amounted to 90, 79, 85, 79 and 86 % for CP, Lys, Met, Thr and Trp, respectively. Overall, SID values ranged from 79 (Lys) to 93 % (Arg and Ile) for indispensable and from 77 (Ala) to 95 % (Pro) for dispensable AA.

**Table 5:** Standardized ileal digestibility (SID) of crude protein (CP) and amino acids (AA; %) and basal ileal endogenous losses (IAA_end_; g/kg dry matter intake) in the barley genotypes (*n* = 68)

Item	SID ± SEM	*P*-value^*^	IAA_end_ ± SEM	*P*-value^**^
CP	90 ± 0.8	<0.001	35.34 ± 9.91	0.001
Indispensable AA				
Arg	93 ± 0.6	<0.001	1.22 ± 0.35	0.001
His	83 ± 0.7	<0.001	0.34 ± 0.16	0.040
Ile	93 ± 0.6	<0.001	1.10 ± 0.22	<0.001
Leu	86 ± 0.5	<0.001	1.29 ± 0.39	0.002
Lys	79 ± 0.9	<0.001	1.08 ± 0.35	0.003
Met	85 ± 0.5	<0.001	0.25 ± 0.09	0.009
Phe	92 ± 0.4	<0.001	1.27 ± 0.21	<0.001
Thr	79 ± 1.0	<0.001	1.02 ± 0.40	0.013
Trp	86 ± 0.7	<0.001	0.38 ± 0.10	0.001
Val	83 ± 0.6	<0.001	0.89 ± 0.35	0.013
Dispensable AA				
Ala	77 ± 1.2	<0.001	1.18 ± 0.55	0.037
Asp	81 ± 1.0	<0.001	1.86 ± 0.64	0.005
Cys	87 ± 0.4	<0.001	0.43 ± 0.11	<0.001
Glu	94 ± 0.4	<0.001	3.44 ± 0.99	0.001
Gly	84 ± 1.4	<0.001	2.05 ± 0.64	0.002
Pro	95 ± 0.7	<0.001	3.35 ± 0.84	<0.001
Ser	91 ± 0.9	<0.001	1.35 ± 0.45	0.004

*P-values of the estimates for the slope of the regression equations; ^**^ *P*-values of the estimates for the intercepts of the regression equation

## Discussion

There were only small variations in the chemical composition of the eight barley genotypes, as indicated by small coefficient of variation, and these variations had only minor impact on SID of AA in these genotypes [7]. The average CP and AA contents of the barley genotypes were within the range of values reported by NRC [30]. Contents of aNDFom and ADFom of the barley genotypes were in the range of NDF and ADF values reported in current feed tables for barley [30]. Contents of NSP were greater than values obtained by Baidoo and Liu [31] for both hulled and hulless barley, whereas contents of *β*-glucan and arabinoxylan were similar to values reported for hulless barley [31, 32].

The IAA_end_ values represented in the present study by the intercept of regression analyses between cAID and dietary contents of CP and AA, were substantially greater than average IAA_end_ obtained from N-free feeding as summarized by Jansman et al. [8] (e.g. for CP 35.3 vs 11.8 g/kg DMI and for Lys 1.1 vs 0.4 g/kg DMI). Similarly, regression analysis revealed also greater IAA_end_ in cereal-based diets compared with N-free feeding in earlier studies (e.g. [33]). These findings have been confirmed in recently published reports by Eklund et al. [9] for rye, Rosenfelder et al. [11] for wheat, and Strang et al. [10] for triticale, where IAA_end_ were considerably greater than average values reported for N-free feeding [8]. Moreover, Fan and Sauer [34], Pedersen et al. [35], and Spindler et al. [3] reported also greater IAA_end_ in barley-based diets determined by the same method of regression analysis as in the present study compared to N-free feeding. Several factors are known to be responsible for the large differences in the endogenous CP and AA outputs among studies. These factors include among others variations in the range of dietary CP levels used for regression analysis [13, 36], the physiological status of the pig [37] but also the use of different methods for estimating IAA_end_ values [38, 39], and contents and types of dietary fiber in the assay diets [39, 40].

According to Fan et al. [12], the closer the graded dietary levels of CP and AA to the origin of the coordinate, the more reliable estimates of IAA_end_ can be obtained. Thus, extrapolation of IAA_end_ from a dietary CP level of 109.1 g/kg DM to zero CP intake may be associated with some uncertainties as also indicated by the large standard error in the present study. On the other hand, all estimates of IAA_end_ obtained in the present study were significantly different from zero, which is in agreement with the results of several other studies, where dietary CP levels of approximately 100 g/kg assay diet resulted in significant estimates of IAA_end_ associated with large standard errors [3, 13, 34, 36, 41, 42]. However, at smaller dietary CP below 50 g/kg, total (IAA_end_ + feed specific) endogenous losses may increase in a nonlinear manner with increasing dietary inclusion level of the assay feed ingredient [43]. Therefore, the range of CP levels in the present study, above this critical level, is more reliable than a wider range, that includes considerably lower CP levels, closer to the coordinate.

Furthermore, feeding of N-free diets or low protein diets over a prolonged period of time can result in increased ileal endogenous AA recovery [37, 44], mainly due to elevated endogenous Pro loss. According to Jansman et al. [8], a Pro content exceeding 30 % of the total quantity of AA in ileal digesta points towards non-physiological conditions. In the present study, a crystalline AA mixture was added on top of the assay diets during the initial 3.5 d of every period as suggested by Pedersen et al. [15] to attenuate any possible effects of the low-protein barley-based assay diets on estimates of IAA_end_. As a result, Pro contributed only 15 % to total protein recovery in ileal digesta of pigs, thus a normal physiological state can be assumed. Therefore it is rather unlikely, that the observed high IAA_end_ in the present study is due to the low protein content of the barley assay diets.

Standardized ileal digestibility of CP and AA in the present study was consistently between 6 and 19 % greater than mean SID of CP and AA reported by Spindler et al. [7], where SID values were determined in the same eight barley genotypes by correcting AID values for IAA_end_ obtained by the N-free alimentation method [8]. Furthermore, compared to tabulated SID values for barley [30, 45], SID values of CP and most AA reported herein were greater, in part close to values for wheat [16] or protein ingredients such as soybean meal [30]. However, greater SID values in the present experiment compared to tabulated values are most likely not caused by differences in chemical composition, as no significant effects of chemical composition on regression analysis could be observed. Similar to the findings of the present work, recent studies with wheat [11], rye [9], and triticale [10] revealed greater SID values by means of the regression method compared to SID values obtained by correction of AID values for IAA_end_. In support of these findings, Spindler et al. [3] and Fan and Sauer [34] reported greater IAA_end_, determined by regression analysis, associated with barley and other cereal grains compared to those obtained by N-free feeding or the use of highly digestible diets. Thus, differences between SID values derived from regression analysis and SID values obtained by correcting AID values for IAA_end_ can mainly be attributed to variations in IAA_end_ values. Estimation of SID of AA by regression analysis requires significant linear relationships between AID and total dietary contents of CP and AA. Therefore, the relative small range of dietary CP levels from 109.1 to 123.8 g/kg DM may result in some uncertainty for the estimates of SID of CP and AA as indicated by large standard errors in the present study. However, highly (*P* < 0.001; except for Gly, where *P* < 0.03) significant linear relationships between AID and total dietary contents of CP and AA were obtained. Moreover, there is evidence, that the range of dietary CP levels had only minor impact on estimates of SID of AA [13].

The use of N-free diets has been accepted as a routine procedure in generating SID of CP and AA in feed ingredients [6]. In particular at low levels of CP in the assay diets, correction of AID values for their IAA_end_ can easily result in an over- or underestimation of SID values due to the substantial correction effect for IAA_end_ at low dietary CP levels, as IAA_end_ have been shown to vary greatly between and within animals and experiments (e.g. [46]). In contrast, the slopes of the linear regression between dietary contents of apparent ileal digestible and total CP and AA provide direct estimates of SID without need for a separate consideration of IAA_end_.

## Conclusion

Basal ileal endogenous losses of CP and AA resulting from the regression between cAID and total dietary contents of AA are considerably greater in cereal grains including barley, compared to reports on IAA_end_ obtained from N-free alimentation. Furthermore, the results of the present study show that SID of CP and AA in cereal grains such as barley determined by regression analysis is substantially greater (up to 14 %) compared to previously reported SID values in feed tables. These differences may be attributed to varying estimates of IAA_end_ among studies. For low-protein feed ingredients including cereals such as barley, the regression method, where SID values are directly obtained from the slope of the linear regression model, may be preferred over correction of AID values for IAA_end_ determined under N-free feeding conditions.

## Abbreviations

AA: Amino acid
ADF: Acid detergent fiber
ADFom: Acid detergent fiber expressed exclusive of residual ash
ADL: Acid detergent lignin
AID: Apparent ileal digestibility
aNDF: Neutral detergent fiber with heat stable amylase and expressed exclusive residual ash
BW: Body weight
cAID: Apparent ileal digestible content
CP: Crude protein
DM: Dry matter
DMI: Dry matter intake
GE: Gross energy
IAA_end_: Basal ileal endogenous losses
N: Nitrogen
NDF: Neutral detergent fiber
NSP: Non-starch polysaccharides
SID: Standardized ileal digestibility
